# Energy Expenditure and Substrate Oxidation in Response to Side-Alternating Whole Body Vibration across Three Commonly-Used Vibration Frequencies

**DOI:** 10.1371/journal.pone.0151552

**Published:** 2016-03-14

**Authors:** Elie-Jacques Fares, Nathalie Charrière, Jean-Pierre Montani, Yves Schutz, Abdul G. Dulloo, Jennifer L. Miles-Chan

**Affiliations:** Laboratory of Integrative Cardiovascular and Metabolic Physiology, Division of Physiology, Department of Medicine, University of Fribourg, Fribourg, Switzerland; University of Rome Foro Italico, ITALY

## Abstract

**Background and Aim:**

There is increasing recognition about the importance of enhancing energy expenditure (EE) for weight control through increases in low-intensity physical activities comparable with daily life (1.5–4 METS). Whole-body vibration (WBV) increases EE modestly and could present both a useful adjuvant for obesity management and tool for metabolic phenotyping. However, it is unclear whether a “dose-response” exists between commonly-used vibration frequencies (VF) and EE, nor if WBV influences respiratory quotient (RQ), and hence substrate oxidation. We aimed to investigate the EE-VF and RQ-VF relationships across three different frequencies (30, 40, and 50Hz).

**Methods:**

EE and RQ were measured in 8 healthy young adults by indirect calorimetry at rest, and subsequently during side-alternating WBV at one of 3 VFs (30, 40, and 50 Hz). Each frequency was assessed over 5 cycles of intermittent WBV (30s vibration/30s rest), separated by 5 min seated rest. During the WBV participants stood on the platform with knees flexed sufficiently to maintain comfort, prevent transmission of vibration to the upper body, and minimise voluntary physical exertion. Repeatability was assessed across 3 separate days in a subset of 4 individuals. In order to assess any sequence/habituation effect, an additional group of 6 men underwent 5 cycles of intermittent WBV (30s vibration/30s rest) at 40 Hz, separated by 5 min seated rest.

**Results:**

Side-alternating WBV increased EE relative to standing, non-vibration levels (+36%, p<0.001). However, no differences in EE were observed across VFs. Similarly, no effect of VF on RQ was found, nor did WBV alter RQ relative to standing without vibration.

**Conclusion:**

No relationship could be demonstrated between EE and VF in the range of 30-50Hz, and substrate oxidation did not change in response to WBV. Furthermore, the thermogenic effect of intermittent WBV, whilst robust, was quantitatively small (<2 METS).

## Introduction

In today’s modern societies sedentariness has become the norm, and amidst increasing recognition of its risks for obesity and cardiometabolic diseases [1], there has been considerable interest in simple methods to enhance energy expenditure (EE) and maximise fat oxidation for body weight control [2, 3]. In this context, whole-body vibration (WBV) has received much attention over recent years. Some 80 years since the first reports of its physiological effects, WBV has become an accepted adjuvant to traditional exercise training programs, with beneficial effects shown in terms of balance, muscle power, and flexibility (for a comprehensive review, see [4]). Moreover, in light of research demonstrating the ability of WBV to evoke muscle activation [5, 6], WBV itself, rather than the physical exercises performed while on the device, is now often promoted as an alternative to more traditional exercise modalities, capable of increasing EE to elicit weight loss [7–10]. As WBV in the absence of concomitant physical activity requires little or no voluntary effort–that is, the device exerts force to cause muscular work–it is of considerable interest not only to individuals with reduced mobility, but also those lacking the impetus for more demanding forms of exercise for weight control. Indeed, exercise performed in combination with WBV has been shown to increase EE more than that without vibration [11–14], albeit rather modestly, with Rittweger estimating the energetic cost of 3 minutes of continuous, side-alternating WBV (*f* = 26 Hz, *A* = 3 mm) to be approximately 10 g fat/h [15]. However, the results of weight loss interventions combining exercise and WBV are equivocal [10, 16–18]. Furthermore, the energy cost of WBV without concomitant exercise has not been comprehensively measured in a well-standardized laboratory setting (e.g. under fasting conditions), nor has it been determined whether this energy cost is influenced by vibration frequency when the participant is simply standing (isometric high-squat) on the device, in the absence of dynamic-eccentric-concentric, or more intense isometric exercise. Furthermore, despite numerous studies of oxygen consumption during WBV [13, 19–22], to-date no measurements of respiratory quotient (RQ; an index of substrate oxidation) without concomitant dynamic exercise have been reported. We therefore aimed to investigate the EE and RQ in response to acute intermittent side-alternating WBV across three commonly-used vibration frequencies (30, 40 and 50 Hz) in a group of young, healthy adults. Based on findings of previous studies [6, 13, 23, 24]), and in particular our own findings of a linear relationship between energy expenditure and low-level intermittent isometric load using leg press [25] we hypothesized that (i) EE would increase during vibration versus non-vibration, and in proportion to vibration frequency (due to increased muscular activity), and (ii) RQ would decrease during vibration versus non-vibration (due to increased activation of oxidative muscles associated with posture and postural stability).

## Materials and Methods

### Subjects

14 young, healthy adult volunteers participated in the present study (Table 1), with a mean (±SEM) age of 25 ± 1 y, weight of 72 ± 4 kg, and body mass index (BMI) of 23 ± 1 kg/m^2^. All subjects were moderately active young adult students (on average, 2 h/week of structured physical activity) recruited through advertisements placed around the University campus, and were all weight-stable, with less than 3% body weight variation in the six months preceding the study. Smokers, pregnant or breast-feeding women, individuals taking medication, and those with any metabolic disease were excluded. Women were only tested during the follicular phase of their menstrual cycle. The study complied with the Declaration of Helsinki and was approved by the Fribourg cantonal ethical review board; all participants gave written consent.

**Table 1:** Subject characteristics.

Subject	Gender	Age	Weight	Height	BMI	Protocol
		(y)	(kg)	(m)	(kg/m^2^)	#
1	F	26	55.5	1.58	22.2	I
2	M	19	93.3	1.86	26.9	I
3	F	27	50.2	1.67	18.1	I, II
4	M	29	67.1	1.82	20.3	I, II
5	F	23	58.6	1.68	20.8	I, II
6	M	25	88.1	1.80	27.1	I, II
7	F	22	58.8	1.69	20.6	I
8	M	32	69.6	1.74	23.0	I
9	M	22	91.6	1.85	26.8	III
10	M	25	80.4	1.85	23.5	III
11	M	21	77.8	1.83	23.2	III
12	M	27	67.6	1.79	21.1	III
13	M	32	74.1	1.71	25.3	III
14	M	23	78.9	1.79	24.6	III
**Average**		25.2	72.3	1.8	23.1	
**SEM**		1.0	3.6	0.0	0.7	

Protocol I: Frequency: 30 Hz, 40 Hz, 50 Hz; Protocol II: protocol 1 repeated on 3 separate days; Protocol III: Frequency: 40 Hz, 40 Hz, 40 Hz. BMI, Body mass index.

### Study Design

Prior to the day of testing, participants visited the laboratory in order to complete a questionnaire regarding their lifestyle and medical history, and to familiarize themselves with the experimental procedure and equipment (including the vibrating platform). All participants were requested to avoid physical activity, caffeine, and dietary supplements in the 24h prior to testing.

**Fig 1:**
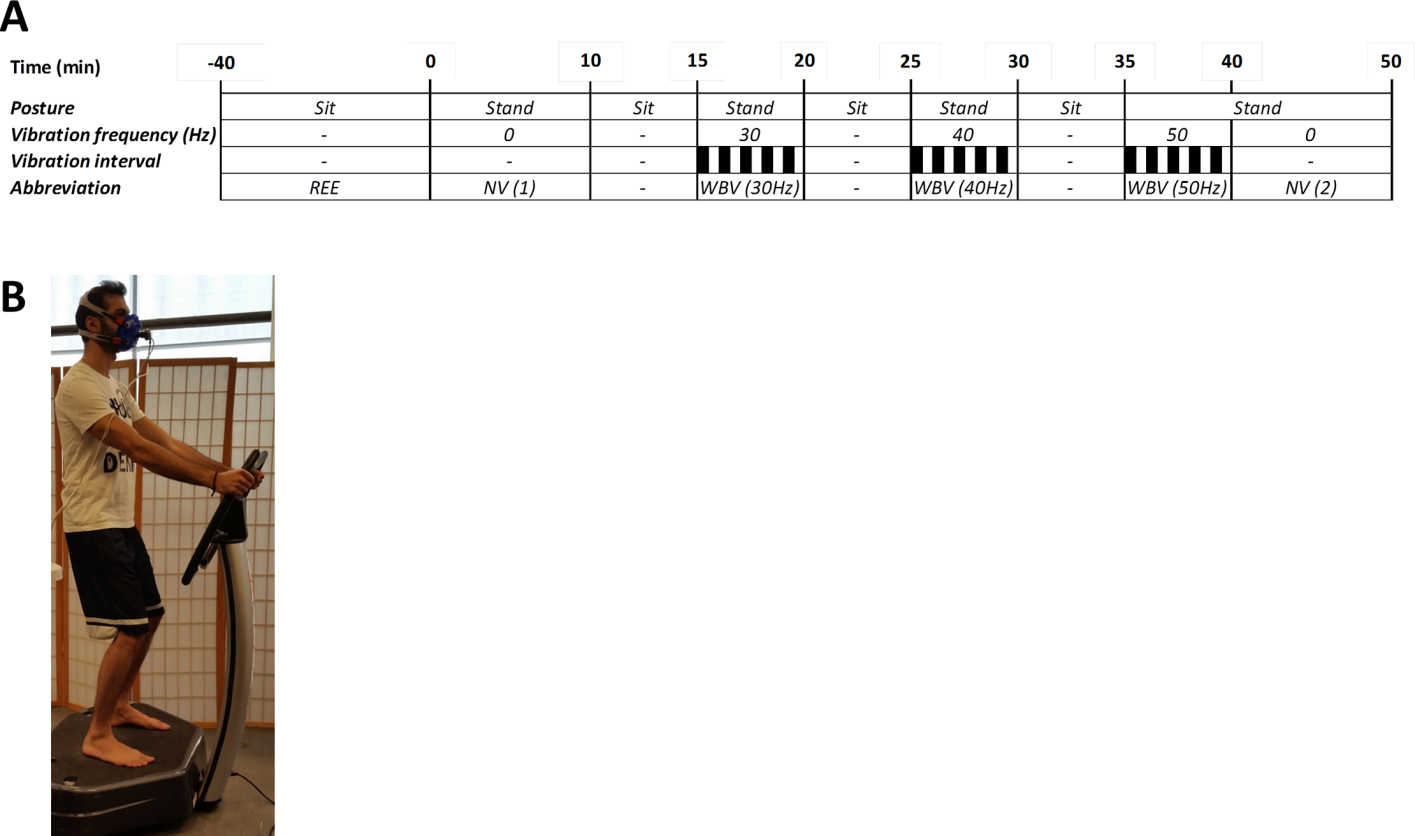
Panel A: Schema showing experimental protocol for intermittent side-alternating whole body vibration (WBV) across varying frequencies. Black bars represent periods of WBV, with each frequency (30, 40 or 50 Hz) assessed over 5 cycles of 30 s vibration and 30 s rest (on the platform), and separated by 5 min rest on a comfortable chair. EE at each vibration frequency was calculated as the integrated mean across the entire 5 min intermittent WBV period (i.e., including measurements obtained during both the 30 s vibration and 30 s rest intervals). Black bars indicate vibration periods. REE: resting energy expenditure while sitting in a comfortable seat (sit); NV: participant standing on platform with no vibration. Panel B: Photo of the position adopted by subjects on the vibrating platform.

Of the 14 participants studied, 8 participants took part in Protocol I, to investigate the effect of side-alternating WBV frequency (30–50 Hz) on EE and RQ (Table 1). The design of this study is shown in **Fig 1A**.

On the day of testing, participants arrived at the laboratory at 8h00 following a 12h overnight fast. After the participants voided their bladder, they were seated on a comfortable chair for measurement of resting parameters for 30–40 minutes.

The participant was then asked to stand, barefoot on a side-alternating vibration platform (Silver Platine, DS’M Creation SA, Bulle, Switzerland), without vibration, for 10 min while cardiometabolic monitoring was continued. This non-vibration standing period was used as a baseline measurement

Following this baseline measurement and a 5 min rest period, during which the participant was seated in a comfortable chair, 3 levels of intermittent vibration frequency (*f*) were tested (30, 40 and 50 Hz) in ascending order. EE, RQ, HR and BR were assessed over 5 cycles of 30 s vibration and 30 s rest (on the platform) at each frequency, separated by 5 min rest on a comfortable chair. After completion of the last 50 Hz cycle, participants remained standing on the platform, with no vibration, for an additional 10 min prior to the completion of the measurements. Each postural transition took less than 1 min, with the measurements during the transition and inter-frequency rest periods excluded from the analyses.

#### Indirect calorimetry

Throughout the experiment, oxygen consumption (VO_2_), carbon dioxide production (VCO_2_), heart rate (HR) and breathing rate (BR) were measured using the Quark CPET indirect calorimeter (Cosmed, Rome, Italy) equipped with a Hans Rudolph silicon facemask. RQ was calculated as the ratio of carbon dioxide produced to oxygen consumed (i.e., RQ = VCO_2_/VO_2_). EE was calculated according to the Weir equation [26]: EE = 5.68 VO_2_ + 1.59 VCO_2_−2.17 N_u_. As short-term urinary collections to assess total nitrogen excretion (N_u_) may not be representative of the protein oxidized during the measurement itself, they were not obtained in this study, and assumed to be 13g/24h; the latter value reflects urinary nitrogen excretion of subjects in post-absorptive (fasted) state [27]. It should be noted that, as described previously [28], this assumption will not significantly influence the relative partition between carbohydrate and fat oxidation determined by indirect calorimetry because (i) the average overnight-fasted RQ of the subjects (~ 0.80) is close to the RQ of protein (~ 0.82), (ii) the proportion of total oxidation derived from protein is small (12–15%) and (iii) in response to the very low intensity exercise (< 2 METS) as in our study here, this proportion is likely to be even smaller.

#### Vibration Protocol

Vibration specifications were verified by securely attaching two triaxial accelerometers (Bioharness BT, Zephyr Technology, MD, USA) to the unloaded vibration platform at approximately the foot placement of the subjects. Peak acceleration and frequency were then measured over 30 s of vibration at each frequency setting. The measured frequency (*f*) from both devices matched that stated by the device manufacturer (i.e., 30, 40 and 50 Hz). Amplitude (*A*) was calculated using the average of the peak acceleration (*a*_*peak*_) values of both devices, where: $A=\;\frac{a_{\mathrm{peak}}}{2\;x\;\pi^{2}\;x\;f^{2}}$. Peak-to-peak amplitude was stated by the manufacturer to be 4 mm, but calculated from average peak acceleration (7.5, 9, and 10 g) as 4, 3, and 2 mm at 30, 40, and 50 Hz, respectively. Intermittent vibration was used so as to limit fatigue and stress, and is in line with the majority of research published thus far and usual fitness-center protocols [4]. During the WBV, the participants stood with their feet shoulder-width apart and weight evenly distributed. Both heel and forefoot in contact with the platform surface, with participants looking forward and holding a non-vibrating hand-rail (as shown in Fig 1B). In order to investigate the energy cost of WBV with minimal voluntary physical exertion, and prevent the transmission of vibration to the upper body, participants were instructed to slightly flex their knees (~10°) so as to avoid discomfort.

### Assessment of repeatability and habituation

A subset of participants (n = 4) repeated the above vibration protocol on 3 days, separated by at least 1 day each, to assess repeatability (Table 1; Protocol II).

In addition, as the frequency sequence in Protocols I and II was fixed (i.e., 30 Hz, 40 Hz, then 50 Hz) we tested for the presence/absence any habituation effect in a separate group of 6 men (Table 1; Protocol III). These subjects underwent a similar protocol to that described above, but with vibration frequency fixed at 40 Hz (rather than changing frequency) for each of the three vibration periods.

### Data and statistical analyses

Sample size for protocols I and III was determined according to the results of *a priori* power analysis (alpha = 0.05; power = 0.8) using the mean (± SD) standing EE and RQ obtained from a previous study of 22 individuals [3], and based on a physiologically relevant increase in EE of 25%, and decrease in RQ of 0.05. *Post hoc* power analyses showed the power for detection to be >90% for the primary variable (EE). Data normality was verified by the Shapiro–Wilk test. The statistical treatment of data was performed using the computer software STATISTIX 8 (Analytical Software, St. Paul, Minnesota, USA). Specifically, to test hypotheses (i) and (ii) repeated-measures ANOVAs were used, followed by Dunnett’s multiple comparison tests (to compare versus baseline EE or RQ), and Tukey HSD All-Pairwise Comparisons Tests (to compare between vibration frequencies). Initially, in order to account for any differences in the dependent variables (EE, RQ, HR, and BR) attributed by gender, ANCOVA analysis (using gender as the covariate) was used; however, as no significant effects of gender were found, data for males and females was pooled for further analysis. Using the data collected from Protocol II, the coefficient of variation (CV) was assessed across the three different frequencies within the same day (inter-frequency, intra-day), as well as at each frequency across 3 separate days (intra-frequency, inter-day). Correlations between the EE and anthropometric characteristics were examined by calculation of Pearson’s correlation coefficient. For all analyses, the significance levels were set to p<0.05, p<0.01, and p<0.001. All data are presented as Mean ± SEM unless otherwise stated.

## Results

### Energy Expenditure

**Fig 2:**
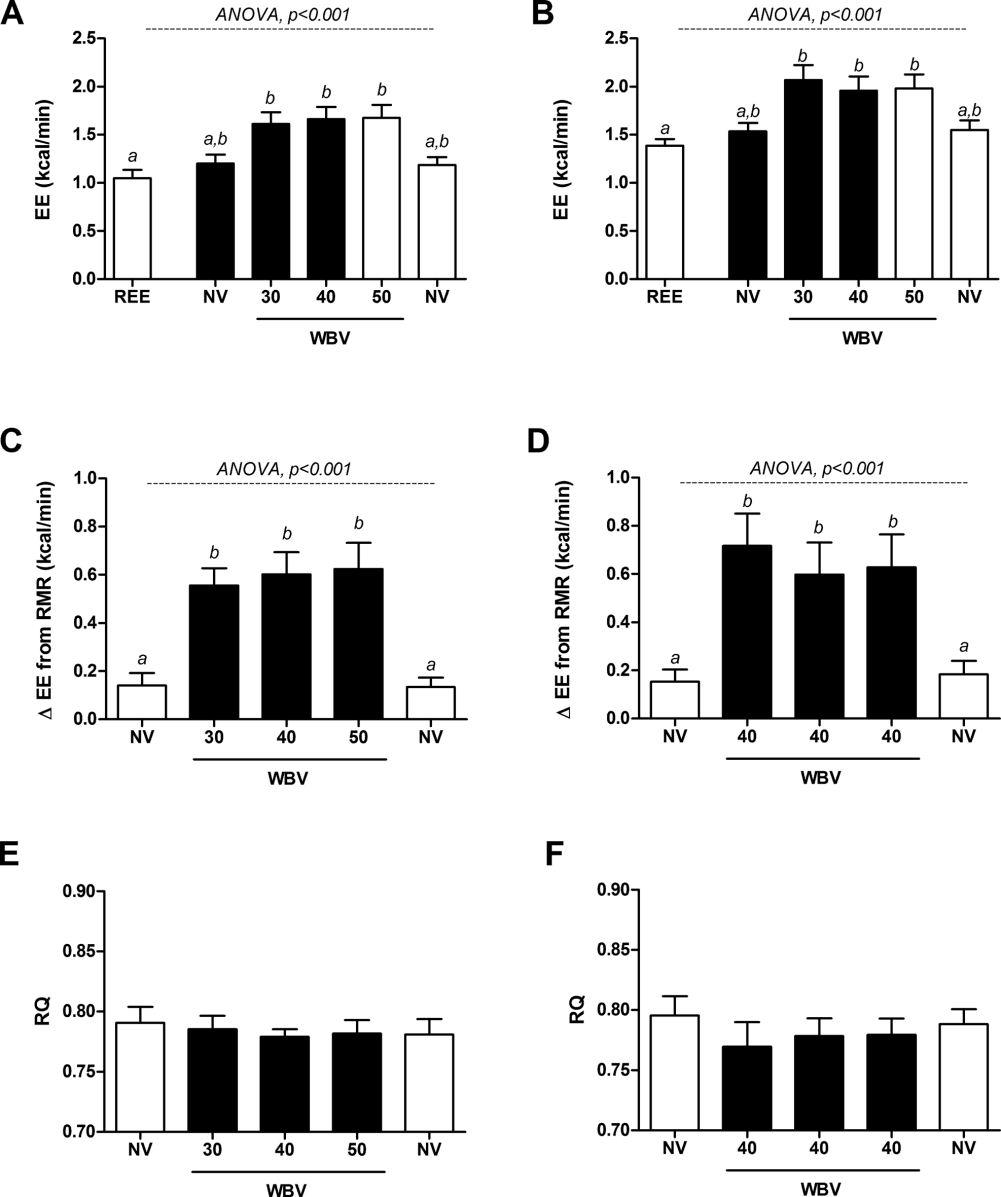
Effects of three frequencies of intermittent side-alternating whole-body vibration (WBV) on energy expenditure (EE) and respiratory quotient (RQ). Left-hand panels (A, C, E) show EE and RQ measured in 8 healthy, young adults across a range of vibration frequencies (30–50 Hz) compared to standing with no vibration. Right-hand panels (B, D, F) show EE and RQ measured across three consecutive vibration periods in 6 healthy, young men at a fixed frequency of 40 Hz. White bars: standing, no vibration (NV); black bars: WBV. Panels A & B: WBV frequencies not sharing letter (*a*,*b*) are different from one another, as assessed by repeated measures ANOVA followed by Tukey HSD All-Pairwise Comparisons Test.

Across the three different frequencies, side-alternating WBV increased EE relative to seated REE (+53%, p<0.001) and to the non-vibration (NV) standing period (+36%, p<0.001), independently of gender. However, as shown in Fig 2A and 2B, no statistically significant differences were observed across vibration frequencies. These results were consistent when considering absolute VO_2_ (+100 ml O_2_/min compared to standing NV period (p<0.001); no effect of vibration frequency) or VO_2_ adjusted for body weight (+1.4 ml O_2_/kg/min compared to standing NV period (p<0.001); no effect of vibration frequency). Additionally, no correlation was found between EE response to vibration at any of the three frequencies tests (versus standing NV period) and any of the measured anthropometric variables (i.e., height, weight or BMI).

**Fig 3:**
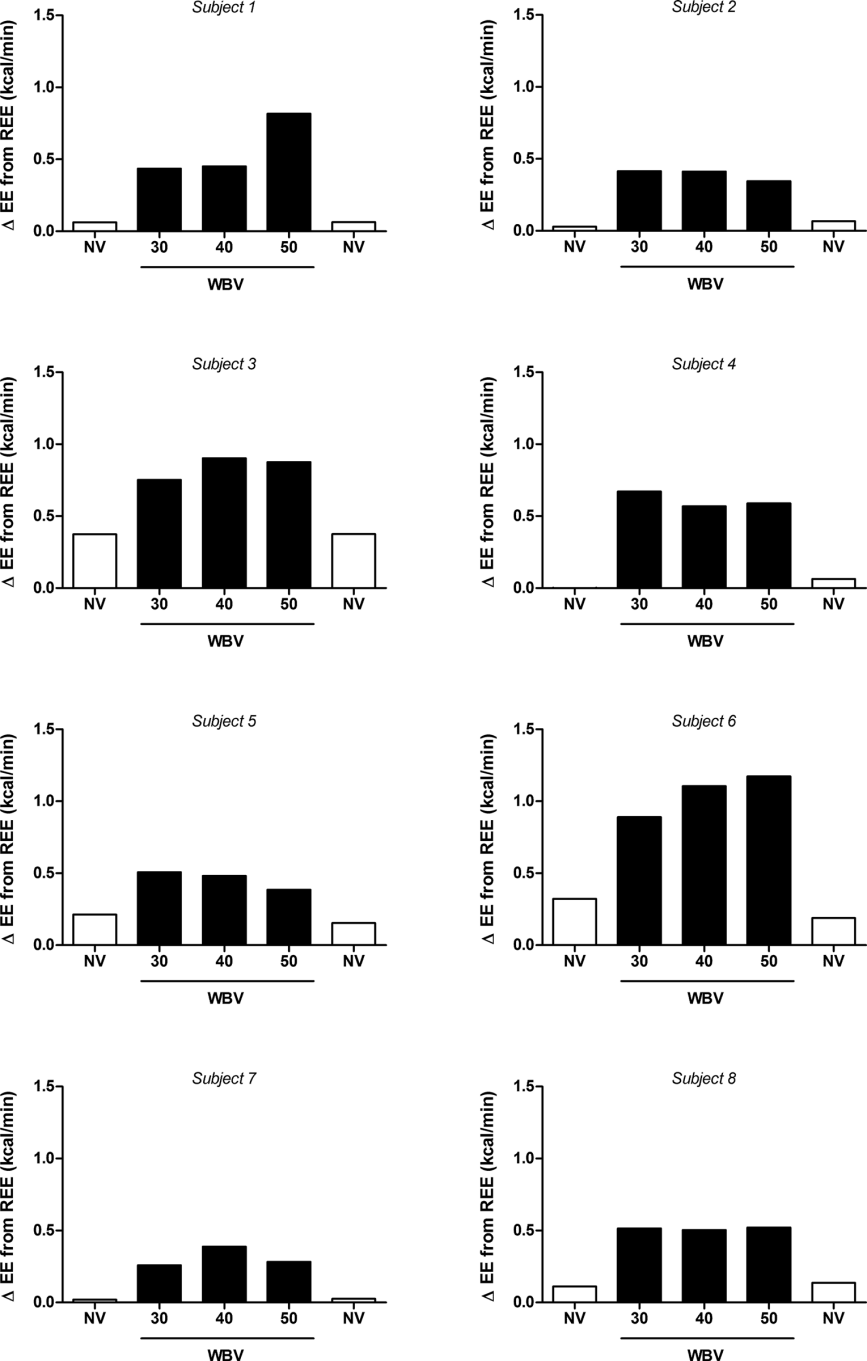
Effects of three frequencies of intermittent side-alternating whole-body vibration (WBV) on energy expenditure (EE) in 8 individual subjects. White bars: standing, no vibration (NV); black bars: WBV.

Individual responses to the WBV protocol are shown in Fig 3, with a positive dose-response observed in only 1 of the 8 subjects (Subject #6). All other subjects showed either no consistent relationship between EE and vibration frequency, or a possible negative dose-response (Subject #5). Overall, when compared to sitting REE, average EE across the SV frequency range was equal to only 1.5 METS (range: 1.2–1.9).

**Fig 4:**
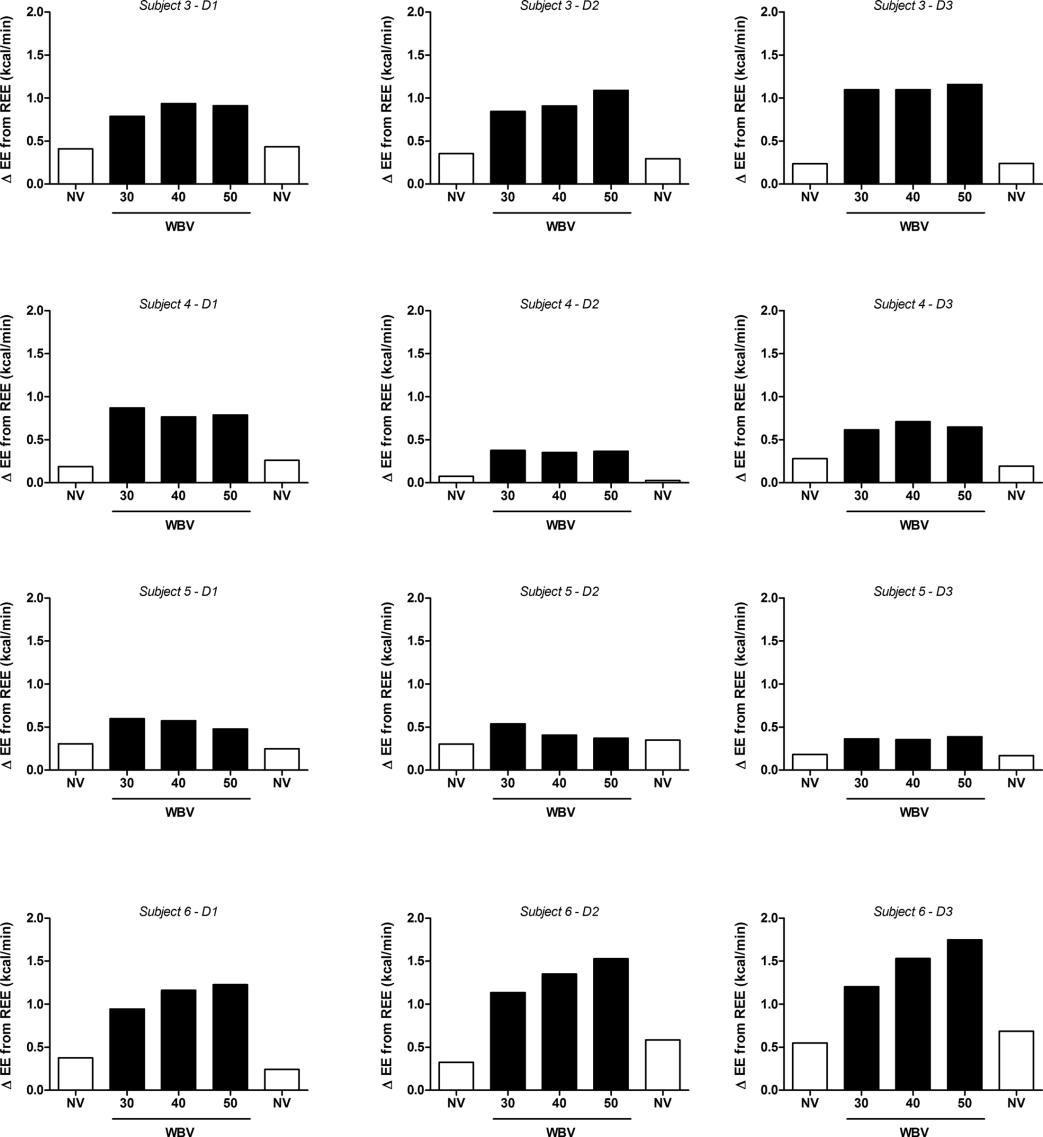
Effects of three frequencies of intermittent side-alternating whole-body vibration (WBV) on energy expenditure (EE) in 4 individual subjects across 3 days (D1-D3). White bars: standing, no vibration (NV); black bars: WBV.

In a subgroup (n = 4) which repeated the protocol on 3 separate days, these results were found to be reproducible, with no significant difference found across vibration frequencies on any of the three experimental days (**Fig 4**).

**Fig 5:**
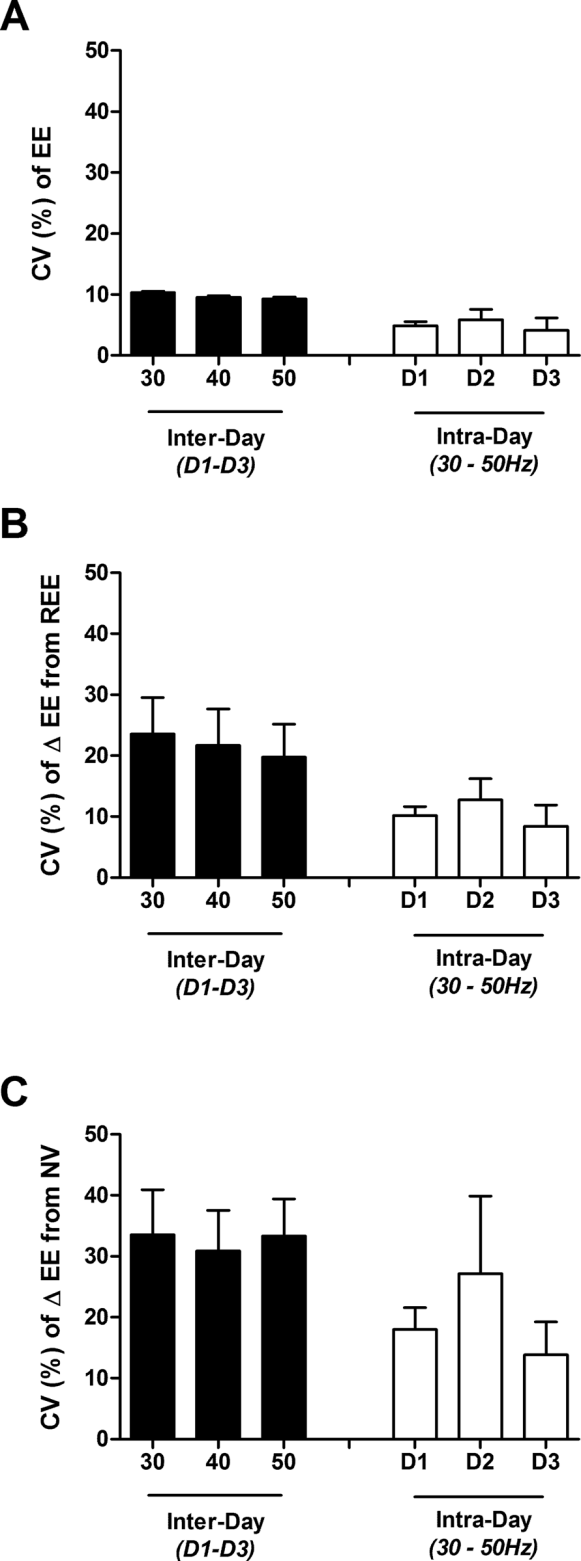
Mean (± SEM) intra- and inter-frequency coefficient of variation (CV %) of energy expenditure in 4 individual subjects across 3 days. Panel A: raw EE; Panel B: delta EE from seated resting EE (REE); Panel C: delta EE from standing with no vibration (NV). Black bars represent mean intra-frequency (inter-day) CV ± SEM, white bars represent mean inter-frequency (intra-day) CV.

The mean within-individual coefficients of variations (CV) in EE during sitting (REE), and standing without vibration (NV) were 2.2, and 7.7, respectively. As shown in **Fig 5**, for each of the three days the variability across the vibration frequencies (intra-day, inter-frequency CV = 4.8, 5.9, and 4.1% for D1, D2 and D3, respectively) was less than that measured across days at each frequency (intra-frequency, inter-day CV = 10.4%, 9.6%, and 9.3% for 30, 40 and 50 Hz, respectively). This observation can also be made in Subject #6, the only subject who appeared to exhibit a consistent dose-response, who displayed 8% variability across the 3 different vibration frequencies, with mean intra-frequency variability of 9%. As also shown in Fig 5, lower inter-frequency variability than intra-frequency variability persisted regardless of considering EE (Fig 5A), delta EE from REE (Fig 5B), or delta EE from NV (Fig 5C).

Within the subjects (n = 6) who underwent 3 consecutive vibrations periods at 40 Hz, EE also increased in response to vibration (+31%, p<0.001) as compared to standing with no vibration (NV; Fig 2B). A comparison between the three successive vibration periods showed that EE during the first vibration period was slightly (0.1 kcal/min), but not significantly higher than the second.

Cardiorespiratory parameters (HR and BR) were measured in a subset of participants (n = 6) undergoing intermittent WBV at 30, 40 and 50 Hz. HR increased during vibration compared to standing NV periods (+3–4 beats/min; p<0.05), with no difference in HR across vibration frequencies (p<0.05). No effects of intermittent WBV or vibration frequency on BR were observed.

### Respiratory Quotient

WBV had no effect on RQ, with no differences found between vibration and standing NV measurements during the 30-40-50 Hz vibration protocol (p = 0.8; Fig 2C), or during the 40-40-40 Hz test of repeatability protocol (Fig 2D).

No subjects reported any adverse side-effects of the WBV protocol at any time during or following the testing period.

## Discussion

### Energy cost of side-alternating WBV

In line with previous reports [11–14], we found an increase in EE with side-alternating WBV, but importantly we found no linear relationship between EE and vibration frequency across 30–50 Hz, indicating an absence of a “dose-response” between these two variables in this commonly-used range of vibration frequencies; the difference in EE across frequencies was small (on average less than 5%). Indeed, variability in EE measured across the 3 different frequencies (30, 40, 50 Hz) within the same day, was smaller than that measured in the same frequency across 3 different days (~5% versus ~10%), indicating that any observed changes in EE between frequencies were within the EE measurement error due to technical (accuracy, precision) and biological (within-subject) variability. Direct in-vitro application of vibration to isolated muscle has been shown to increase ATP turnover [29], as has WBV in combination with moderate-intensity isometric exercise (with ATP turnover in calf muscle increasing 60% versus the control, non-vibration condition). The latter was not detectable under natural perfusion [30]. However, whilst WBV has become a widely used tool within both the consumer fitness industry and clinical research over recent years, the effects of WBV, in its own right (or under conditions of minimal physical exertion), on whole-body response in terms of EE and substrate oxidation is largely unexplored. Rittweger investigated the energy demand of side-alternating WBV and found it to increase proportionally to increasing vibration frequency [13] in the range of 18–34 Hz. However, this latter study was conducted using continuous vibration after a 10 min cycling and stretching warm-up, with no control of absorptive state, and subjects were given the opportunity to slowly walk around during inter-vibration rest periods; all of which may have influenced the measured energetic response to vibration. Furthermore, the range of vibration frequencies used was lower, and the amplitude higher (5 mm) than those employed in the present study. In contrast, our measurements were conducted under strictly-standardised, overnight fasting conditions, and using intermittent vibration. Indeed, our finding may be at least partially explained by considering the effects of vibration on skeletal muscle. Fratini *et al*. [31] have previously reported that maximum muscle activation will occur when WBV frequency is matched to the muscle’s natural resonance frequency–which in the case of the soft tissue of the lower limbs ranges from 5 to 65 Hz [32, 33]. It is therefore possible that, in our subjects, different muscles or muscle groups were activated at different frequencies such that while different patterns of activation occurred across vibration frequencies, the net effect in terms of energetic cost (activity x muscle mass), was unchanged. It is important to note that with increasing vibration frequency we measured a small decrease in the amplitude of vibration of the unloaded platform used in this study. As such, acceleration load, highlighted in recent studies [34–36] as potentially the most important parameter for optimising muscle activity during WBV, did not change proportionally to increasing frequency, and may have confounded any EE-VF dose-response. Conversely, it could be argued that the range of frequencies tested here was too narrow to observe a relationship with EE, although the range chosen here was in line with that used by others (as reviewed by Cochrane [4]), and covers the common frequency ranges of commercially-available devices used within the consumer fitness industry. In addition, a small but not significant habituation effect was observed in the test of repeatability (protocol II), with EE during the first 40 Hz vibration period slightly higher than the second (0.1 kcal/min, ~5%), although this difference was not statistically significant and unlikely to have influenced the main finding of the study.

### Implications for physical activity and weight control

An important implication of our study pertains to claims by some that WBV increases EE in a similar fashion as moderate walking at 4.5 km/h, usually defined as 3–4 METS [2], and that this energy cost may be elevated by increasing vibration frequency [13, 20]. Furthermore, it has been suggested, based upon oxygen consumption values assessed over 3 min of continuous vibration at 26 Hz, that the energy cost of WBV would amount to fat loss of approximately 10 g fat/h [13]- which would correspond to an increase in EE of 1.5 kcal/min and about 2.5 METS. In contrast to these claims, EE measured during the intermittent side-alternating WBV protocol used in the present study averaged just 1.5 METS. We chose to use intermittent rather than continuous vibration in order to align with the majority of previously published vibration studies [2] and to avoid problems associated with fatigue (particularly amongst non-athletic participants) and stress, which may confound measurements of EE. Thus, the protocol chosen better represents the usual scenario found in the consumer setting, in which WBV is often promoted and employed as a tool for body weight management. However, while there was a substantial inter-individual variability in terms of response (1.2–1.9 METS), the energetic cost of a typical 30-min intermittent vibration session, within a common-used range of frequencies, would be unlikely to exceed 30 kcal. Indeed, even doubling the energy cost of intermittent side-alternating WBV measured in the current study (so as to approximate the energy cost of continuous vibration), would equal just 2 METS. The inter-individual variability in EE response to WBV observed in the present study may represent that in muscle activation [6] and hormonal response [37] observed by others. Indeed, in the present study, only one subject showed a tendency toward a positive dose-response between vibration frequency and EE, indicating individual rather than vibration characteristics may play a larger role in determining energetic response.

Interestingly, the lack of a relationship between EE and vibration frequency in most individuals in our study contrasts with recent findings [35, 36] that during vibration, EMG activity of several specific leg muscles was dependent upon the acceleration loads within the frequency window (30–50 Hz) used in the present study. There are several explanations for this apparent discrepancy. *First*, specific leg muscle EMG activity response may not necessarily translate into a proportional increase in whole-body EE. *Second*, not all leg muscles may show the EMG dependency on the acceleration load, as recently shown by Giminiani et al. [36]. *Third*, one may argue that the EE response to vibration frequency may be highly individual, a contention that would seem to be supported by our data indicating highest EE being reached at 50 Hz in some individuals, but at 30 or 40 Hz in others (Fig 3). However, our inter-day repeatability study revealed that in any individual, the highest EE response was not observed at the same vibration frequency on each of the three different measurement days (Fig 4). Taken together, the small differences in EE across vibration frequency are most likely due to background noise in EE measurements resulting from methodological and intra-individual variability.

### Effects of side-alternating WBV on substrate utilization

Whilst other researchers have investigated the effect of WBV (usually in combination with exercise) on oxygen consumption, little data is available regarding substrate utilization. One study has reported a decrease in fat oxidation (estimated from RQ) during squatting with vibration versus without, but the effect of WBV *per se* was not investigated [12]. In the present study, we found no change in RQ during WBV compared to standing without vibration. While the longer-term effects of WBV on substrate oxidation have not been explored in humans, identical frequencies of WBV (30–50 Hz) has been shown to reduce body fat accumulation and serum leptin levels in female rats, without alterations in food intake or muscle mass [38]. Furthermore, several studies in humans have shown decreases in fat mass in individuals undergoing WBV training [8, 16–18]. However, all these studies have involved vibration in combination with varying forms of cardiovascular or resistance training, and thus interpretation of the effect of WBV *per se* is difficult. In contrast, our finding of no effect of intermittent WBV on RQ is supported by two studies investigating the acute hormonal effects. They observed no notable change in endocrine profile (i.e., no indication of lipolysis) in response to WBV, with the exception of a transient decrease in plasma glucose (most likely due to increased utilization by muscle), and increased norepinephrine (most likely due to stimulation of the peripheral sympathetic neurons by standing) [37, 39]. Results from other studies investigating the effect of WBV on growth hormone, insulin-like growth factor-1, and testosterone secretion are equivocal [40–43]. Interestingly, a recent meta-analysis indicates that while WBV (particularly at lower frequencies than those tested here) may increase peripheral blood flow, it does not alter skeletal muscle oxygenation [44], which may further support the lack of observed increase in fat oxidation.

### Limitations

The present study has several limitations which should be considered in the interpretation of our findings. *Firstly*, the results of the present study may have been confounded by alterations in vibration amplitude. Estimation of amplitude by accelerometry indicated that, as discussed earlier, vibration amplitude appeared to decrease with increasing frequency. Furthermore, it would be expected that this amplitude could be further altered by platform loading–that is, the body weight of the subject. Subjects in the present study varied considerably in body weight (range: 50–93 kg), although, no correlation was found between this body weight (or any other anthropometric characteristics) and EE response to WBV. *Secondly*, our intention was to study WBV under conditions of minimal voluntary physical activity. As such subjects were instructed to flex their knees sufficiently to maintain balance and comfort, and prevent the transmission of vibration to the upper body [45]. However, the angle of knee flexion (~10°) was not strictly-controlled, and therefore the degree of isometric work performed by each subject, while minimised, may not have been identical. *Third*, only young, healthy subjects were recruited for the present study as a proof-of-principle, in order to investigate the energetic cost of WBV in the absence of excess adiposity and/or metabolic disease. Therefore, it warrants further investigation as to whether or not overweight or obese individuals respond differently to increasing vibration frequency in the context of increasing energy expenditure and/or fat oxidation for treating/preventing obesity. In conclusion, the 36% increase in EE during standing with WBV versus without, and the relatively low intra-individual variability observed, indicates a robust but quantitatively small thermogenic effect of WBV (<2 METS). However, within this commonly-used WBV frequency range (30–50 Hz), no energetic “dose-response”, or any effect on substrate utilization was observed. Nevertheless, whilst the acute effects are minimal (<30 kcal per 30 minutes, or 60 kcal/h), this small increase in EE cumulated over time may indeed assist weight management, particularly in the prevention of weight gain or regain in healthy individuals, but requires prospective studies.
