# Correction: Energy Expenditure and Substrate Oxidation in Response to Side-Alternating Whole Body Vibration across Three Commonly-Used Vibration Frequencies

**DOI:** 10.1371/journal.pone.0163822

**Published:** 2016-09-22

**Authors:** Elie-Jacques Fares, Nathalie Charrière, Jean-Pierre Montani, Yves Schutz, Abdul G. Dulloo, Jennifer L. Miles-Chan

There are errors in the labelling of the x-axis of Fig 2B. The x-axis label should read “REE, NV, 40, 40, 40, NV”. There is no error with the data itself, the statistics, or the description of the results. Please see the corrected Fig 2 here.

**Fig 2:**
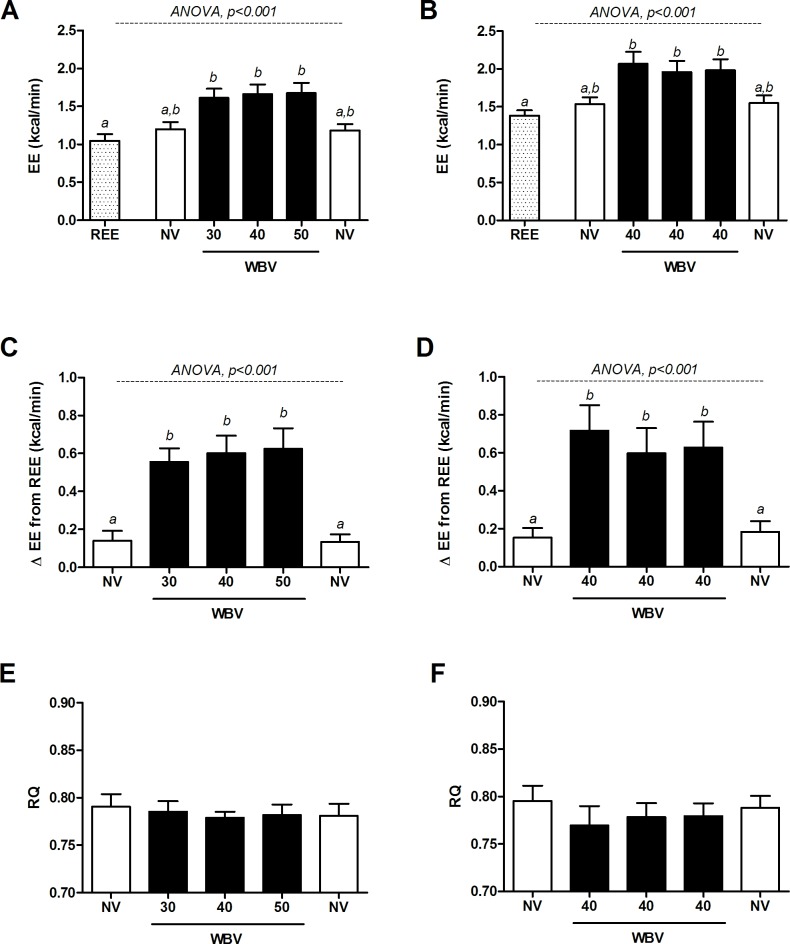
Effects of three frequencies of intermittent side-alternating whole-body vibration (WBV) on energy expenditure (EE) and respiratory quotient (RQ). Left-hand panels (A, C, E) show EE and RQ measured in 8 healthy, young adults across a range of vibration frequencies (30–50 Hz) compared to standing with no vibration. Right-hand panels (B, D, F) show EE and RQ measured across three consecutive vibration periods in 6 healthy, young men at a fixed frequency of 40 Hz. White bars: standing, no vibration (NV); black bars: WBV. Panels A & B: WBV frequencies not sharing letter (*a*,*b*) are different from one another, as assessed by repeated measures ANOVA followed by Tukey HSD All-Pairwise Comparisons Test.

Also, the following minor corrections to Fig 2 in-text citations are needed:

The second sentence of the first paragraph of the Energy Expenditure subsection in the Results should read: However, as shown in as shown in Fig 2A and 2C no statistically significant differences were observed across vibration frequencies.

The first sentence of the fifth paragraph of the Energy Expenditure subsection in the Results section should read: Within the subjects (n = 6) who underwent 3 consecutive vibrations periods at 40 Hz, EE also increased in response to vibration (+31%, p <0.001) as compared to standing with no vibration (NV; Fig 2D).

The first sentence of the first paragraph of the Respiratory Quotient subsection of the Results section should read: WBV had no effect on RQ, with no differences found between vibration and standing NV measurements during the 30-40-50 Hz vibration protocol (p = 0.8; Fig 2E), or during the 40-40-40 Hz test of repeatability protocol (Fig 2F).
